# Tin and Gold in Combination for Filling Teeth

**Published:** 1890-08

**Authors:** W. D. Miller

**Affiliations:** BERLIN.


ARTICLE III.

### TIN AND GOLD IN COMBINATION FOR FILLING TEETH.

BY PROFESSOR W. D. MILLER, BERLIN.

Some men claim that tin and gold in combination is as good as tin, no better and no worse. Dr. Bonwill recently expressed himself on this subject, but his remarks show that he knows no more of the merits of this combination than those who know very little.

It is remarkable that so few men in America have taken a fancy to this method, when such men as Drs. Abbot of Berlin, and Jenkins of Dresden, after nearly a quarter of a century of experience, say that in many cases it is preferable to all other fillings. The trouble seems to have been that in America men have not known how to differentiate as to the best locations in which to use tin and gold.

The proper way to prepare these two metals for use as a filling is to lay a sheet of either number four, five, or six non-cohesive gold foil on a sheet of number four tin foil, extra weight, and roll into ropes. In filling, work the material as one would non-cohesive gold. The manipulation is as easy, if not more so.

No special instruments are needed, though I devised a set which I believe are still kept in stock by the S. S. White Company. I depend chiefly on Nos. 1, 2, 5, 6, and 7 of this set. This, however, should be born in mind: that whatever the shape of the instrument, the point should be square, not round, because packing must be done sidewise.

The advantages which may be claimed for tin and gold are:

1. Exceeding softness and adaptability.

2. The ease and rapidity with which it may be manipulated, thus greatly simplifying the filling of large cavities for persons of extreme nervous temperament.

3. Indifference to moisture. It may sometimes occur that slight moisture may creep over the filling during the operation. This will not interfer with success and need give no uneasiness. I have made perfect fillings in mouths deluged with moisture where it has been impossible to dam up the saliva.

4. The consolidation of the material. An electrical decomposition, and then redisposition of the tin on the gold occurs, thus producing a solid mass.

5. This material invariably hugs the walls of the cavity and the margins.

6. It is a poor conductor of heat.

The disadvantage is that it assumes a color varying from a dull gray to black. It has neither therapeutic nor antiseptic action.

There are certain places where tin and gold seem specially indicated. These are: (1) Grinding surface cavities in lower molars; (2) molars only partly erupted, the use of the dam being impossible; (3) poorly developed six-year molars; (4) buccal cavities near or under the gum margin. (The cements or gold are practically worthless in these cases); (5) small grinding cavities in temporary teeth; (6) teeth in which it is impossible or inadvisable to remove all the decay. Dip the first pieces in carbolic acid, and further decay will be prevented.

In many large cavities where it is desired that the surface of the filling shall be of gold, much time may be saved by filling with tin and gold about two thirds of the cavity, completing the filling with gold alone. In small, deep, or narrow cavities in grinding surfaces, or where the decay ramifies into the sulci, a better filling may be made beginning with tin and gold, and covering with gold, than could be made with gold alone. Much less of the tooth substance need be removed in such cases if the combination is used, owing to its softness and adaptability.

It sometimes occurs that there are overhanging edges of enamel which are strong and therefore should not be removed. This leaves deep undercuts difficult to fill properly with gold. Tin and gold should be used along the walls and under the overhanging enamel, and then gold wedged in the centre. There is no danger of such a wedge coming out, as it becomes a part of the whole mass under the electrical change already alluded to.

In crown cavities, where the pulp is nearly approached, tin and gold should be used on account of the low conductivity.

Tin and gold is of inestimable value in large distal proximate cavities. Such cavities, whether they open into the grinding surface or not, and especially in bicuspids, if filled with gold, will remain in good condition for three or four years at the outside. With tin and gold perfect fillings may be made, which will save the teeth indefinitely. Apply a matrix, place a piece of the rope against the buccal edge of the lower border of the cavity, and condense it. Follow with another piece against the lingual side, then a piece in the centre to wedge the others, then mallet. Continue in this way till the filling is well in sight, and complete the operation with gold. In upper incisors having frail walls, cover the labial walls with non-cohesive gold and pack against it with the combination. Another valuable use for the material is where otherwise good gold fillings are leaking about the edges. They may be repaired with this combination, and will stand well.

DISCUSSION.

*Dr. Stockton.*—I have used tin and gold for a great many years. I began it perhaps as a matter of economy, using it in charity cases early in my career. I can show now some of those fillings twenty-five years old, still in good order. Dr. Miller alludes to one class of cases where he tells us that we can cut out as little as possible of the cavity. Cut out as much as possible, say I. Prepare all cavities so that you can see to the bottom. There are more failures from too little than from two much cutting of cavities. The trouble with too many of us is that we do not control our patients, but allow them to control us. Recently a lady came into my office, suffering from pericementitis caused by a dead pulp. I took up my engine and began to drill into the tooth. The patient cried out, "Stop, doctor; I will not allow you to cut away my tooth when there is no cavity." I laid down my instruments and said: "Go elsewhere, madam; I can do nothing for you. You have no confidence in me." In my office and in my practice I am master. That is as it should be. Then we can select our own materials for filling teeth, and cut away cavities or not as the need of each case indicates. Dr. Miller makes one mistake. He says that tin and gold has no therapeutic action. If it saves a tooth it has a therapeutic action. Anything which will stop decay in a tooth is therapeutic.

*Dr. Gordon White.*—I have had made for me some copper foil, which I am using under gold fillings as Dr. Miller uses tin. Thus far I am much pleased with the result.

*Dr. Barton.*—I do not believe that tin and gold will unite as is claimed. I have removed fillings in which no such union has taken place. I have such a filling in my mouth and no union has occurred. Dr. Stockton is right: anything is therapeutic which protects the tooth. I think tin and gold no better than soft gold, though easier to work.

*Dr. R. C. Young.*—It is a well known law of electrolysis, that at the positive poles the hydrates of metals are deposited and at the negative gases are liberated, and thus metals are polarized. In this combination under discussion the tin is positive to the gold; therefore we have the salt of tin deposited, which may be absorbed between the dentinal tubuli.

Heat is transmitted by induction. This accounts for the low conductivity of the tin and gold filling. The heat passing through the gold meets the tin, which proves an obstacle. Much has been said and written about the disintegration of the cervical edges under fillings of gold. In those cases the dentine is negative to the gold; therefore a hydrogen gas is liberated at that point. Hydrogen is the active principle of all acids which destroy dentine. This is prevented when we use tin and gold, because of the salt which is deposited.

*Dr. Patrick, of Belleville.*—I have the profoundest respect for Professor Miller. If I had not known that he wrote this paper I should never have suspected it. It is the worst paper I ever heard from him. I used tin and gold for forty years. Then I grew wise, and I have not used it for twenty. I used it from a motive of economy.—*Dental Mirror.*

